# Genetics and epigenetics of obesity

**DOI:** 10.1016/j.maturitas.2011.02.018

**Published:** 2011-05

**Authors:** Blanca M. Herrera, Sarah Keildson, Cecilia M. Lindgren

**Affiliations:** a Wellcome Trust Centre for Human Genetics, University of Oxford, Roosevelt Drive, Oxford OX3 7BN, United Kingdom; b Oxford Centre for Diabetes, Endocrinology and Metabolism University of Oxford, United Kingdom

**Keywords:** BMI, body mass index, WC, waist circumference, WHR, waist:hip ratio, T2D, type-2-diabetes, LD, linkage disequilibrium, CNV, copy number variants, PWS, Prader–Willi syndrome, QTL, quantitative trait loci, SNP, single nucleotide polymorphism, Genetic, Epigenetic, Fat distribution, Obesity, Methylation, Imprinting

## Abstract

Obesity results from interactions between environmental and genetic factors. Despite a relatively high heritability of common, non-syndromic obesity (40–70%), the search for genetic variants contributing to susceptibility has been a challenging task. Genome wide association (GWA) studies have dramatically changed the pace of detection of common genetic susceptibility variants. To date, more than 40 genetic variants have been associated with obesity and fat distribution. However, since these variants do not fully explain the heritability of obesity, other forms of variation, such as epigenetics marks, must be considered.

Epigenetic marks, or “imprinting”, affect gene expression without actually changing the DNA sequence. Failures in imprinting are known to cause extreme forms of obesity (e.g. Prader–Willi syndrome), but have also been convincingly associated with susceptibility to obesity. Furthermore, environmental exposures during critical developmental periods can affect the profile of epigenetic marks and result in obesity.

We review the most recent evidence for genetic and epigenetic mechanisms involved in the susceptibility and development of obesity. Only a comprehensive understanding of the underlying genetic and epigenetic mechanisms, and the metabolic processes they govern, will allow us to manage, and eventually prevent, obesity.

## 1 Introduction

Overweight and obesity are becoming more widespread with global projections of more than 2.16 billion overweight and 1.12 billion obese individuals by 2030 [1]. This clearly presents a worldwide clinical and public health burden, associated with social and personal criticism. It is also correlated with an increased risk of type-2-diabetes (T2D), cardiovascular disease, cancer and mortality [2,3]. Despite intensive research, current efforts to reduce obesity by diet, exercise, education, surgery and drug therapies are failing to provide effective long-term solutions to this epidemic.

At an individual level, obesity occurs when abnormal amounts of triglycerides are stored in adipose tissue and released from adipose tissue as free fatty acids (FFA) with detrimental effects [4]. Excess fat accumulation occurs when energy intake exceeds energy expenditure, although individuals respond differently to this imbalance owing to genetic predisposition. Twin studies estimate heritability of body mass index (BMI) to be 40–70% in children and adults [5–7], and other anthropometric measures of obesity and regional fat distribution [skinfold thickness, waist circumference (WC) and waist:hip ratio (WHR)] show similar heritability [5–12]. Furthermore, there are ethnic differences in obesity; admixture mapping studies demonstrate that obesity correlates closely with the percentage of ancestry derived from ethnic groups with elevated prevalence [13,14]. The goal of obesity research is to elucidate pathways and mechanisms that control obesity and to improve prevention, management and therapy.

Here we review recent advances in identifying factors contributing to obesity susceptibility. We focus on:

(a) recent successes in identification of genetic variation affecting obesity trait susceptibility;
(b) emerging evidence connecting epigenetic (heritable changes which affect gene function but do not modify DNA sequence) events with obesity.

We discuss the impact of recent findings in these two areas and their joint potential to enhance understanding of obesity susceptibility mechanisms and aetiology.

## 2 The identification of susceptibility loci for obesity

Until 2006, the main approaches used to track down common variants influencing obesity, involved either hypothesis-free genome-wide linkage mapping in families with multiple obese subjects or association studies within ‘candidate’ genes using case–control samples or parent–offspring trios. The former suffered from being underpowered for any sensible susceptibility models, as linkage is best placed to detect variants with high penetrance. As far as we can tell, common variants with high penetrance do not contribute substantially to risk of common forms of obesity and few, if any, robust signals have emerged from such efforts [15,16]. The latter candidate-gene association approach has historically been compromised by difficulties in selecting credible candidates. Selection was typically based on hypotheses about biological mechanisms putatively involved in obesity pathogenesis but, as the function of much of the genome is poorly characterized, it remains almost impossible to make fully informed decisions. In addition, all too often these candidate-gene studies were conducted in sample sets far too small to offer confident detection of variants with the range of effect sizes that are now known to be realistic. With hindsight, it is easy to appreciate why these approaches yielded few examples of genuine obesity–susceptibility variants.

Consequently, over the last two decades, efforts in identifying and replicating genetic variants predisposing individuals to common forms of obesity were largely characterized by slow progress and limited success, in sharp contrast to the successful gene identification in monogenic and syndromic forms of obesity [15]. The most recent edition of the “Human Obesity Gene Map” gives an excellent overview of this; it lists 11 single gene mutations, 50 loci related to Mendelian syndromes relevant to human obesity, 244 knockout or transgenic animal models and 127 candidate genes, of which slightly less than 20% are replicated by 5 or more studies [15]. A total of 253 quantitative trait loci (QTL), for different obesity-related phenotypes, have been reported from 61 genome-wide linkage scans and of these, only ∼20% are supported by more than one study [15].

Over the past three years it has become possible, from technical and economic perspectives, to undertake hypothesis-free GWA testing in samples of sufficient size to generate convincing association results. The advent of the GWA approach was the result of three components. The first was the human genome sequence which subsequently enabled cataloguing genome-sequence variation. Secondly, the International HapMap Consortium (http://www.hapmap.org) [17] taught us that, in non-African-descent populations, extensive correlations (linkage disequilibrium, LD) between neighbouring single nucleotide polymorphisms (SNPs) constrain the number of independent genetic tests required to survey the genome, such that ∼80% of all common variation can be sampled using ∼500 000 carefully selected SNPs [18,19]. Lastly, novel genotyping methods address the challenges of massively parallel SNP-typing at high accuracy and low cost [20]. The GWA approach has been hugely successful in identifying loci harbouring common forms of obesity (as defined by anthropometric measures: BMI, WC and/or WHR) susceptibility genes and hereto results from a total of 15 ‘high-density’ GWAs (i.e. ≥300 000 SNPs, offering genome-wide coverage >65%) have been published (Table 1). These studies combined, have yielded over 50 loci associated with obesity (*p*-values <5 × 10^−8^ in genotyped and imputed data sets, or <5 × 10^−7^ in directly genotyped data only) (Table 2).

The first gene unequivocally associated to common, non-syndromic obesity, *FTO* (fat mass and obesity associated) [21], was initially identified as a result of a GWA of T2D [22]. While it was the second strongest associated locus, the association was completely abolished when adjusting for T2D. The association of the *FTO* region to obesity explains ∼1% of BMI heritability, such that adults homozygous for the risk allele, have a 2–3 kg higher weight compared to non-risk allele homozygous [21]. Interestingly, *FTO* is reported to operate on fat mass and was suggested to encode a 2-oxoglutarate-dependent nucleic acid demethylase involved in regulation of food intake [23]. In parallel, it was reported to be involved in decreased lipolytic effect in adipocytes [24]. It is unclear whether the association effect acts through *FTO* or the adjacent *FTM* gene and the precise role of the *FTO* locus in obesity needs further investigation.

With reports of the first, robust dichotomous trait associations [25] and the discovery of *FTO* [21,26,27], came the realization that the effect sizes detected would be smaller than anticipated and that successful analysed would require larger sample sizes than previously considered. This insight catalyzed large-scale international collaboration and meta-analyses of existing data. Through the first collaboration in obesity research, a strong association was detected between SNPs located 188 kilobases (kb) downstream from the melanocortin 4 receptor gene (*MC4R*) and BMI (Tables 1 and 2 [28]). In parallel, it was reported to be associated with WC in individuals of Indian Asian or European ancestry (Table 1 [29]). The risk variant has subsequently been associated with higher energy and fat intake [30] and the increased BMI reported in children, is consistent with early onset obesity caused by *MC4R* mutations [31]. Larger GWA meta-analysis, through the Genetic Investigation of Anthropometric Trait (GIANT) Consortia and deCODE (Table 1) followed reporting 8 novel obesity loci, as well as confirming the *MC4R* and *FTO* associations (Table 2 [32]). Several of the likely causal genes in the associated regions are highly expressed or known to act in the central nervous system (CNS), suggesting, as in rare monogenic forms of obesity, the role of CNS pathways in predisposition to overall obesity [33].

A few smaller GWAs [34–36] focused on other forms of obesity (early onset, extreme obesity and/or morbid adult obesity) and replicated *FTO*, *MC4R* and *TMEM18* BMI-associations. These studies also identified four novel associations (Table 2). Cotsapas et al. [35] observed nominal evidence of association for 7 of the 13 loci previously reported to influence BMI [32–34]. This suggests that variants influencing BMI might also contribute to more severe forms of obesity, which represents the extreme, of the phenotypic spectrum rather than a distinct condition, although, this needs confirmation in appropriately powered studies.

Recently, a large-scale meta-analysis of 249 769 individuals confirmed 14 of these previously identified obesity susceptibility loci and identified 18 novel loci associated with BMI and overall adiposity (Table 2) [37]. As a consequence of the increased power of this study, new signals with lower minor allele frequencies and smaller effect sizes, compared to previously identified variants, were discovered. These results suggest that genome-wide association studies are only the first step in the identification of the causal variants that play a role in common, overall obesity and weight regulation and further insights into the underlying biological mechanisms and pathways will be needed for the effective treatment and management of these traits [37].

While no consistent association has been shown between height and BMI-associated variants as a group, three of the loci (*MC4R*, *RBJ*–*ADCY3*–*POMC* and *MTCH2*–*NDUFS3*) show individual associations with height [37]. The BMI-increasing alleles of the variants near *POMC* and *MC4R* were associated with decreased and increased height respectively, and this is analogous to the effects of severe mutations in *POMC* and *MC4R* on height and weight [31,38].

### 2.1 Genetics of fat distribution

As described above, efforts to identify common and rare variants influencing BMI and risk of obesity have emphasized a key role for neuronal regulation of overall obesity [21–24,26–30,32–35], but until recently provided few clues to processes responsible for variation in central obesity and fat distribution [29,39,40]. Measures of central and general adiposity are highly correlated: BMI has an *r*^2^ ∼ 0.9 with WC and ∼0.6 WHR. Also, WC and WHR are correlated with measures of intra-abdominal fat, measured by magnetic resonance imaging in obese women (*r*^2^ ∼ 0.6 and 0.5, respectively). Clinically, central obesity is associated with susceptibility to T2D, cardiovascular disease and increased mortality [3]. Evidence indicates that individual variability in patterns of fat distribution involve local, depot-specific and body-shape processes, which are probably independent of the mechanisms that control overall energy balance and general obesity. First, anthropometric measures of central adiposity are highly heritable [41] and, after BMI correction, heritability estimates remain high (∼0.6 for WC and ∼0.45 for WHR) [10,11,42]. Second, substantial gender-specific differences in fat distribution, reflect specific genetic influences [43,44]. Third, inherited lipodystrophies, which are monogenic syndromes, demonstrate that DNA variants can have specific effects on the development and/or maintenance of specific regional fat-depots and body shape [45]. Hereto, five GWA studies of central obesity (WC and WHR) have been published [29,39,40,44,46] (Table 1) and have identified 17 novel common obesity loci. The associations of three of these loci (*MSRA*; *TFAP2B* and *NRXN3*, Table 2) to WC appear to be mediated through BMI [36,37,39,44], implying their involvement in overall adiposity. The remaining 14 loci, however, showed significant associations with WHR, after correction for BMI, suggesting their role in body fat distribution, independent of overall obesity. Interestingly, some of these loci showed strong sex-specific effects in women [39,44] and in a study by Heid et al. [44], the WHR-increasing allele was shown to be associated with an increase in WC for 14 loci in women, but only six men. This evident heterogeneity between men and women across many of these WHR loci is a reflection of the sex-specific genetic effects driving individual patterns of body fat.

## 3 Epigenetics and obesity

Epigenetics is loosely defined as the study of heritable changes which affect gene function without modifying the DNA sequence [47]. The maintenance of epigenetic marks through generations is poorly understood and the notion of their transmission is contentious [48]. Epigenetic marks are tissue specific and include DNA methylation and histone modifications which mediate biological processes such as imprinting. As many imprinted genes are growth factors, or regulators of gene expression controlling growth, imprinting disorders often feature obesity as one of their clinical characteristics.

Genomic imprinting determines expression of alleles according to their maternal or paternal origin [49] and establishes a balance between the expression of the parental alleles influencing growth [50], resulting in counteracting growth effects of paternal and maternal genomes [51]. In addition to growth, imprinted genes are also involved in differentiation, development, viability and metabolic functions [52]. Two main clusters of genomic imprinting are known in humans: a region at 11p15 containing several imprinted genes including *IGF2*, *INS*, *KCNQ10T1* (*LIT1*) (paternally expressed) and *H19*, *KCNQ1*, *CDKN1C*, *PHLDA2*, *KVLQT1* (maternally expressed) [53]. The second cluster at 15q11–q12 contains at least 7 imprinted genes, including; *MKRN3*, *MAGEL2*, *NDN*, *SNURF*–*SNRPN* (paternally expressed) and *UBE3A*, *ATP10A* (maternally expressed) [54].

Failures in imprinting which result in obesity by altering expression of growth and cellular differentiation factors can arise due to numerous genetic events: translocation, inversion, duplication, paternal disomy and hyper/hypo-methylation. For instance; paternal deletion or uniparental disomy at 15q11–q13, results in Prader–Willi syndrome (PWS). A syndrome characterized by severe (sometimes life threatening), early onset obesity caused by hyperphagia (due to dysfunction in the satiety centre [55]). Moderate obesity appears in Albright hereditary osteodystrophy (AHO), due to disruption of imprinting at the *GNAS* gene (20q13.11 [50]).

### 3.1 Mediators of genomic imprinting

#### 3.1.1 DNA methylation

**Fig. 1:**
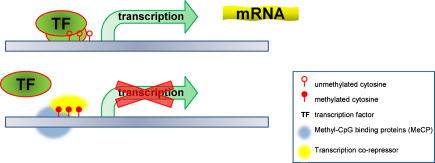
CpG methylation and regulation of gene expression. Unmethylated or hypomethylated DNA (usually in the promoter region) allows binding of the transcription factors (TF) and other regulatory mechanisms which results in transcription and mRNA production. Methylated DNA (bottom panel) obstructs binding of the TF, and in some cases might recruit methyl-CpG binding proteins and other transcription co-repressors, blocking access of the transcription enzymes and resulting in gene silencing.

Genomic imprinting is mediated by DNA methylation as exemplified in the *H19* and *IGF2* loci [56]. Methylation is a widespread feature of the genome, and is obtained through the addition of a methyl group (CH3) to a cytosine positioned next to a guanine nucleotide (CpGs), usually in regions with a high presence of CpG dinucleotides (>60%). Methylation in a promoter region results in the repression (silencing) of gene expression [57], this effect may be achieved by a number of mechanisms including: obstructing access to transcription factors/activators and recruitment of co-repressors (like histone deacetylases) which alter chromatin structure [58] resulting in failure to initiate transcription (Fig. 1).

#### 3.1.2 Histone modifications

DNA in cells is packaged as chromatin in a “beads on a string” configuration. A length of 147 DNA base pairs wraps around a core histone octamer (a tetramer of histones H3 and H4, flanked by two H2A–H2B dimmers) and these nucleosome “beads” are separated by DNA “strings” between 20 and 60 base pairs. Linker histones (H1/H5) occupy the exit and entry of the DNA into the histone, these structures are in turn coiled into a compact helical “closed” configuration [59]. Packaging DNA in this manner allows its efficient storage and is paramount for regulation of gene expression, as the “closed” configuration does not allow access to transcriptional enzymes. Post translational modification of the histones by: H3 and H4 hyperacetylation in the promoter [60], methylation of the lys4 and lys36 of histone H3 open the structure and allow gene expression. In contrast, methylation of lys9, lys20 and lys27 on H3 [60,61]) and ubiquitination of H2A present at high density CpG promoters [62] result in gene silencing “closed configuration”. Further complexity is achieved by the level of methylation, so mono-, bi- or tri-methylation may also effect the control of gene expression [63].

**Fig. 2:**
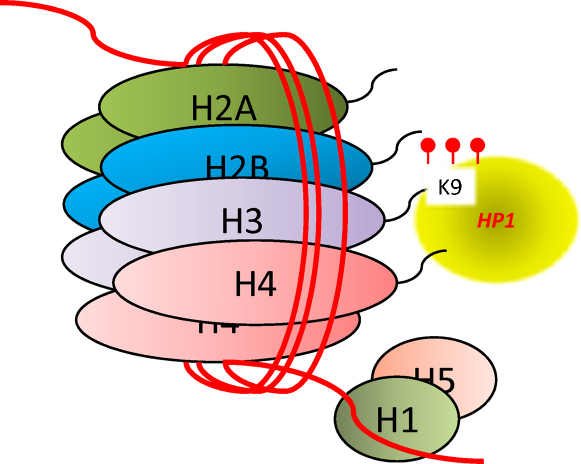
Histone modification. This simplified diagram of a nucleosome shows a histone octamer “bead” surrounded by a DNA strand and try-methylation at lysine-9, this kind of modification exemplifies modifications found at promoter regions of silenced genes.

A feature of both methylation and histone modifications is that they are both tissue specific and can vary with age (and developmental stage). Therefore, in order to place findings in an appropriate context, it is of paramount importance that evaluation of epigenetic factors be carried out on suitable tissues extracted at specified times [64,65] (Fig. 2).

### 3.2 Epigenetic changes introduced during early development may increase the risk of obesity

Although birth weight (BW) is an imperfect summary index of growth, however, it has been widely used as a proxy for foetal nutrition and intrauterine growth. Although better measurements of foetal growth exist, BW measurements are used because they are non-intrusive, easily obtained and often found in birth records, which enabled its use as in retrospective studies linking BW with adult onset diseases [66]. Many studies focus on the hypothesis that early environmental influences induce epigenetic variation, thereby permanently affecting metabolism and chronic disease risk. Specifically, for obesity, it has been shown that obese mothers tend to have obese children [67], it has been shown that clinical intervention to cause maternal weight loss can have a positive effect on reducing risk of obesity in the offspring [68]. The mechanisms by which nutritional challenges affect the risk of disease in later life are poorly understood. However, evidence indicates that the establishment of the epigenome can be affected by environmental factors during critical developmental periods [69]. Possible disturbances of methylation may arise during foetal development due to lack of availability of dietary methyl donors [70–72]. Potential interactions between the environment and epigenetic mechanisms mediating the expression of genes associated with increased BMI and adiposity, may also be possible as suggested for; the *FTO* locus is a DNA-demethylase enzyme [23], the *MC4R* gene which has reduced methylation following long-term exposure to a high fat diet [73], the *PPARγ* protein which interacts with histone acetyltransferases [58] during adipogenesis and on the effect of diet on methylation of *POMC* [74] and *Leptin* [75].

With this in mind, it is important to consider the effect of assisted reproduction technology on epigenetics and subsequently on obesity susceptibility. Despite small numbers, increased incidence of PWS, when compared to background population prevalence, has been shown [76] and subtler adverse effects may only become apparent later on in life. We are currently gaining more insight through the use of high-throughput sequencing methods for the detection of DNA methylation patterns, and chromatin immunoprecipitation (ChIP) used to detect histone modifications and new integrated genomics-based technologies which are currently being developed by the human epigenome project (http://www.epigenome.org) initiative.

## 4 Remaining challenges

### 4.1 Identifying novel loci

Despite successes in susceptibility loci identification for obesity through the first wave of GWAs, the combined effect of the loci explains only 2–3% of the inherited contribution to obesity risk.

### 4.2 Collaborative studies to for larger GWA meta-analysis

The GIANT consortium is finalising the next wave of GWA meta-analysis for obesity related traits incorporating >100 000 samples. This increase in sample size should attain sufficient power to allow identification of common variants with even smaller effect sizes than hereto. Also, conditional analysis using the previously identified variants will reveal whether some of the already identified loci contain more than one independent association with obesity, which would be an additional source of unexplored variation contributing to the missing heritability.

### 4.3 Obesity susceptibility associated to rare-low-frequency variants

The obesity risk alleles that have been identified so far by GWAs are quite common (27–91%) in European populations but the current approaches are limited in the range of minor allele frequencies (MAF) that are detected due to the range of allele frequencies (>5% MAF) present on current genotype arrays. The 1000 genomes project (http://www.1000genomes.org/) is designed to catalogue low and rare frequency variants and this will lead to the design of new genotype arrays including wider spectra of allele frequencies.

### 4.4 Copy number variations

Copy number variants (CNVs) are hard to detect due to technical constrains but recently [74], two complementary approaches have delivered the first examples of CNVs associated to obesity. The SNPs associated with obesity at the *NEGR1* locus are in strong LD with a nearby CNV [32], tagging a 45-kb deletion polymorphism which is a candidate causal-variant at this locus. Recently by analysing SNP genotype arrays that are enriched in CNV information and applying novel CNV detecting algorithms on these arrays deletions on chromosome 16p11.2 have been reported to be associated with extreme, highly penetrant obesity [77,78]. Further applications of these approaches in larger sample sets should allow a better understanding of total CNVs contribution to overall variation in obesity susceptibility.

### 4.5 Characterization of associated loci and causal variants

It is not clear how much of the remaining variation will be uncovered by the aforementioned steps. Beyond identifying more susceptibility loci, finding the causal gene(s) and variant(s) in each locus will be necessary to allow the detailed molecular and physiological investigations that are necessary to determine the mechanisms and pathways through which these contribute to obesity susceptibility and ultimately allow this knowledge to be translated into better prediction and treatment options.

### 4.6 Integration of genetic and epigenetic information

Genetic and epigenetic factors are intimately intertwined, as epigenetic marks and DNA modifications are the direct consequence of sequence-specific interactions between proteins and DNA [79]. The completion of the human epigenome project will increase our understanding of the genetic and epigenetics underlying cellular homeostasis and the integration of this knowledge with the known environmental risks to obesity, must be applied so that it may eventually be exploited and manipulated to appease the obesity epidemic.

## 5 Conclusions

The current obesity epidemic is clearly not of genetic origin per se, but due to unfavourable changes in lifestyle and environment (the ‘obesogenic’ environment). The obesogenic environment has different effects on different individuals in the same environment, highlighting an underlying, inherited susceptibility to obesity and fat-distribution. For more than a decade, the genetics underlying common forms of obesity have remained elusive although the advent of the GWA approach has started to deliver robust associations to obesity. The epigenetic contribution to common forms of obesity are still largely unknown but, from rare syndromes and animal models we conclude that it is likely that both genetic and environmental effects on epigenetics will in turn be associated with obesity. We have started to identify an emerging pattern of effects acting through CNS, suggesting that a component of an individual's response to the obesogenic environment is partly neurobehaviourally driven. There is also some evidence of effects acting more peripherally in the adipose tissue. Despite the success of GWAs in obesity loci identification, we still only explain a low fraction of the inter-individual variation of obesity. Extensive work including identification of more obesity susceptibility loci, a better understanding of the gene(s) through which the effect is executed, as well as further molecular and physiological characterization of the associated genes, is now necessary before any of these findings will lead to any useful therapeutic interventions.

## Contributors and their role

All the authors were involved in the drafting, editing and approval of this manuscript.

Blanca M. Herrera and Sarah L. Keildson were involved in the construction of the tables and figures.

## Competing interests

None.

## Provenance and peer review

Commissioned and externally peer reviewed.

## Figures and Tables

**Table 1:** Overview of GWA scans or meta-analysis thereof for obesity phenotypes.

Reference	Study name (if any)	Number of samples in discovery cohort	Ancestry of discovery cohort	Phenotype
Frayling et al. [20]	WTCCC	1924	Europeans	BMI – quantitative analysis
Scuteri et al. [26]	Sardinia	4741	Europeans	BMI – waist circumference (WC) quantitative analysis
Chambers et al. [28]	LOLIPOP	2684	Indian Asians	Insulin resistance and related quantitative phenotypes
Loos et al. [27]	–	16 876	Northern European	BMI – quantitative analysis
Heard-Costa et al. [36]	The CHARGE consortium	31 373	Europeans	WC – quantitative analysis
Lindgren et al. [35]	The GIANT consortium	38 580	Europeans	WC and waist:hip-ratio (WHR) – quantitative analysis
Cotsapas et al. [33]		775 cases and 3197 unascertained controls	Europeans	Extreme obesity/BMI
Meyre et al. [32]		1380 and 1416 age-matched normal-weight control	Europeans	Early onset and morbid adult obesity
Thorleifsson et al. [31]	DeCODE	37 347	Europeans + African Americans	BMI – quantitative analysis
Willer et al. [30]	The GIANT consortium	32 387	Europeans	BMI – quantitative analysis
Hinney et al. [79]		487 extremely obese young cases and 442 healthy lean controls	Europeans	Extreme obesity/BMI
Scherag et al. [34]		453 extremely obese young cases and 435 healthy lean controls	Europeans	Extreme obesity/BMI
Cho et al. [41]	KARE	8842	Asian	BMI, WHR – quantitative analysis
Heid et al. [43]	MAGIC	77 167	European	WHR – quantitative analysis
Speliotes et al. [37]		123 865	European	BMI – quantitative analysis

**Table 2:** The 54 loci associated to anthropometric obesity phenotypes.

Closest gene(s)	Chromosomal location	Phenotype	Associated lead SNP(s)	Proposed molecular or cellular function	Additional phenotypes	References
*TBX15*–*WARS2*	1p12	WHR	rs984222	Transcription factor involved in adipocyte and specific adipose depot development	Implicated in Cousin syndrome	Heid et al. [43]
*PTBP2*	1p21.3	BMI	rs1555543	–		Speliotes et al. [37]
*NEGR1*	1p31	BMI	rs2815752, rs3101336, rs2568958	Neuronal outgrowth		Thorleifsson et al. [31], Willer et al. [30], Speliotes et al. [37]
*TNNI3K*	1p31.1	BMI	rs1514175	–		Speliotes et al. [37]
*DNM3*–*PIGC*	1q24.3	WHR	rs1011731	Dominant, negative mutations in DNM enzymes promote GLUT6 and GLUT8 transporters to adipocyte cell surface in rats.		Heid et al. [43]
*SEC16B*, *RASAL2*	1q25	BMI	rs10913469	–		Thorleifsson et al. [31], Speliotes et al. [37]
*LYPLAL1*; *ZC3H11B*	1q41	WHR	rs2605100	Encodes protein thought to act as triglyceride lipase and is upregulated in subcutaneous adipose tissue in obese patients		Lindgren et al. [35], Heid et al. [43]
*SDCCAG8*	1q43–q44	BMI	rs12145833	–		Scherag et al. [34]
*FANCL*	2p16.1	BMI	rs887912	–		Speliotes et al. [37]
*RBJ*–*ADCY3*–*POMC*	2p23.3	BMI	rs713586	–	Rare *POMC* mutations cause human obesity	Speliotes et al. [37]
*TMEM18*	2p25	BMI	rs6548238, rs2867125, rs4854344, rs7561317, rs11127485	Neural development	Associated with T2D	Willer et al. [30], Thorleifsson et al. [31], Scherag et al. [34], Speliotes et al. [37]
*ZNRF3*–*KREMEN1*	2q12.1	WHR	rs4823006	–	Kremen1 protein forms a complex with LDL receptor-related protein 6	Heid et al. [43]
*LRP1B*	2q22.2	BMI	rs2890652	–	*LRP1B* deletions seen in several types of human cancers	Speliotes et al. [37]
*GRB14*	2q24.3	WHR	rs10195252	–	Associated with triglyceride and insulin levels. *GRB14*-deficient mice exhibit increased body weight	Heid et al. [43]
*ADAMTS9*	3p14.1	WHR	rs6795735	Important for spatial distribution of cells in embryonic development	Associated with T2D	Heid et al. [43]
*NISCH*–*STAB1*	3p21.1	WHR	rs6784615	Interacts with insulin receptor substrate		Heid et al. [43]
*CADM2*	3p21.1	BMI	rs13078807	–		Speliotes et al. [37]
*ETV5* (locus with three genes, strongest association in *ETV5*)	3q27	BMI	rs7647305	–		Thorleifsson et al. [31], Speliotes et al. [37]
Gene desert; *GNPDA2* is one of three genes nearby	4p13	BMI	rs10938397	–	Associated with T2D	Willer et al. [30], Speliotes et al. [37]
*SLC39A8*	4q24	BMI	rs13107325	–		Speliotes et al. [37]
*FLJ35779*	5q13.3	BMI	rs2112347	–		Speliotes et al. [37]
*ZNF608*	5q23.2	BMI	rs4836133	–		Speliotes et al. [37]
*CPEB4*	5q35.2	WHR	rs6861681	Regulates polyadenylation elongation		Heid et al. [43]
*TFAP2B*	6p12	WC, BMI	rs987237	–		Lindgren et al. [35], Speliotes et al. [37]
Locus containing *NCR3*, *AIF1* and *BAT2*	6p21	BMI	rs2844479, rs2260000, rs1077393	–	Associated with weight, not BMI	Thorleifsson et al. [31]
*VEGFA*	6p21.1	WHR	rs6905288	Involved in vascular development. Key mediator of adipogenesis	*VEGFA* variants nominally associated with T2D	Heid et al. [43]
*NUDT3*–*HMGA1*	6p21.31	BMI	rs206936	–		Speliotes et al. [37]
*PRL*	6p22.2–p21.3	BMI	rs4712652	–		Meyre et al. [32]
*LY86*	6p25.1	WHR	rs1294421	Plays a role in recognition of lipopolysaccharide	Associated with asthma	Heid et al. [43]
*RSPOS*	6q22.33	WHR	rs9491696	Promotes angiogenesis and vascular development	Oncogene in mouse mammary epithelial cells	Heid et al. [43]
*NFE2L3*	7p15.2	WHR	rs1055144	–		Heid et al. [43]
*MSRA*	8p23.1	WC, BMI	rs7826222, rs17150703	–		Lindgren et al. [35], Scherag et al. [34]
*LRRN6C*	9p21.3	BMI	rs10968576	–		Speliotes et al. [37]
*PTER*	10p12	BMI	rs10508503	–		Meyre et al. [32]
*MTCH2* (locus with 14 genes)	11p11.2	BMI	rs10838738	Cellular apoptosis		Willer et al. [30], Speliotes et al. [37]
*BDNF* (locus with four genes, strongest association near *BDNF*)	11p14	BMI	rs4074134, rs4923461, rs925946, rs10501087, rs6265	*BDNF* expression is regulated by nutritional state and *MC4R* signalling	Associated with T2D. Individuals with WAGR syndrome with *BDNF* deletion have BMI >95th percentile. *BDNF* knockdown in mouse hypothalamus causes hyperphagia and obesity	Thorleifsson et al. [31], Speliotes et al. [37]
RPL27A	11p15.4	BMI	rs4929949	–		Speliotes et al. [37]
*ITPR2*–*SSPN*	12p21.1	WHR	rs718314	–	Mice lacking *ITPR2* and *ITPR3* exhibited hypoglycaemia and lean body type	Heid et al. [43]
*HOXC13*	12q13.13	WHR	rs1443512	Transcription factor important in cell spatial distribution in embryonic development		Heid et al. [43]
*FAIM2* (locus also contains *BCDIN3D*)	12q13	BMI	rs7138803	Adipocyte apoptosis		Thorleifsson et al. [31], Speliotes et al. [37]
*C12orf51*	12q24	WHR	rs2074356	–		Cho et al. [41]
*MTIF3*–*GTF3A*	13q12.2	BMI	rs4771122	–		Speliotes et al. [37]
*PRKD1*	14q12	BMI	rs11847697	–		Speliotes et al. [37]
*NRXN3*	14q31	WC, BMI	rs10146997	–		Heard-Costa et al. [36], Speliotes et al. [37]
*MAP2K5*	15q23	BMI	rs2241423	–		Speliotes et al. [37]
*SH2B1* (locus with 19–25 genes)	16p11.2	BMI	rs7498665, rs8049439, rs4788102, rs7498665	Neuronal role in energy homeostasis	*Sh2b1*-null mice are obese and diabetic	Willer et al. [30], Thorleifsson et al. [31], Speliotes et al. [37]
*GPRC5B*	16p12.3	BMI	rs12444979	–		Speliotes et al. [37]
*MAF*	16q22–q23	BMI	rs1424233	Transcription factor involved in adipogenesis and insulin–glucagon regulation		Meyre et al. [32]
*FTO*	16q22.2	BMI	rs9939609, rs6499640, rs8050136, rs3751812, rs7190492, rs8044769, rs1558902	Neuronal function associated with control of appetite	Associated with T2D	Frayling et al. [20], Scuteri et al. [26], Chambers et al. [28], Willer et al. [30], Thorleifsson et al. [31], Meyre et al. [32], Scherag et al. [34], Speliotes et al. [37]
*NPC1*	18q11.2	BMI	rs1805081	Intracellular lipid transport	*NPC1*-null mice show late-onset weight loss and poor food intake. *NPC1* interferes with function of raft-associated insulin receptor signalling	Meyre et al. [32]
*MC4R*	18q22	BMI	rs17782313, rs12970134, rs17700144	Hypothalamic signalling	Haplo-insufficiency in humans is associated with morbid obesity. *MC4R*-deficient mice show hyperphagia and obesity	Willer et al. [30], Thorleifsson et al. [31], Meyre et al. [32], Loos et al. [27], Chambers et al. [28], Scherag et al. [34], Speliotes et al. [37]
*KCTD15*	19q13.11	BMI	rs11084753, rs29941	–		Willer et al. [30], Thorleifsson et al. [31], Speliotes et al. [37]
*QPTCL-GIPR*	19q13.32	BMI	rs2287019	Encodes incretin receptor	Associated with fasting and 2-h glucose	Speliotes et al. [37]
*TMEM160*	19q13.32	BMI	rs3810291	–		Speliotes et al. [37]
